# A qualitative study on utilization of vulnerability assessment tool for heatwave-related health adaptation intervention in Australia

**DOI:** 10.1093/heapro/daaf221

**Published:** 2025-12-19

**Authors:** Patrick Amoatey, Grace Arnot, Aklilu Endalamaw, Nicholas J Osborne, Zhiwei Xu, Dung Phung

**Affiliations:** School of Public Health, Faculty of Health, Medicine and Behavioural Sciences (HMBS), The University of Queensland, 288 Herston Road, Herston, QLD 4006, Australia; Institute for Health Transformation, Faculty of Health, Deakin University, 1 Geringhap Street, Geelong, VIC 3220, Australia; School of Public Health, Faculty of Health, Medicine and Behavioural Sciences (HMBS), The University of Queensland, 288 Herston Road, Herston, QLD 4006, Australia; School of Public Health, Faculty of Health, Medicine and Behavioural Sciences (HMBS), The University of Queensland, 288 Herston Road, Herston, QLD 4006, Australia; School of Population Health, University of New South Wales, 55 Botany Street, Randwick, NSW 2031, Australia; European Centre for Environment and Human Health (ECEHH), University of Exeter Medical School, Peter Lanyon Building 12, Penryn, Cornwall, TR10 8RD, United Kingdom; School of Medicine and Dentistry, Griffith University, Parklands Drive, Southport, Gold Coast, QLD 4222, Australia; School of Public Health, Faculty of Health, Medicine and Behavioural Sciences (HMBS), The University of Queensland, 288 Herston Road, Herston, QLD 4006, Australia

**Keywords:** heatwave vulnerability, interview study, thematic analysis, state professionals, Australia

## Abstract

Heatwave-health vulnerability index (HVI) map is an important tool for mitigating heatwave. However, the perspectives of heatwave-health management professionals (HMPs) about heatwave vulnerability have not been examined. This study aims to understand HMP's perception and experiences of applying the HVI tool for heatwave-related health adaptation and mitigation. A semi-structured interview with HMPs was conducted between June and October 2024 across the Australian state agencies. We conducted an online interview, which lasted ∼31 minutes. Participants were asked to share their perspectives about the HVI tool, its policy applications, challenges, and recommendations. Twenty HMPs, the majority (75%) within 40–59 years old, 10 female (50%), and with a median (range) heat-health work experience of 6 (2–11) years participated in the study. The HMPs were from Health (50%), Climate/Environment (15%), Emergency Services (10%), Meteorology (10%), and City Councils (15%). The five themes identified were the following: (i) HMPs have moderate levels of awareness of heatwave vulnerability, particularly in the context of locating the hottest areas, (ii) HVI is considered an important tool for heatwave planning, public information, and urban design, (iii) adherence to traditional heat management strategies, low national awareness, and resourcing problems are perceived as key barriers to a national HVI, (iv) the current HVI is faced with methodological inconsistencies, limited understanding, and low confidence among policymakers, and (v) HMP are well positioned to evaluate HVI and encourage broader engagement of the index in Australia. This study offers critical insight for improving policy awareness and enhances the evaluation of the HVI through a multidisciplinary approach.

Contribution to Health Promotion

- This article discusses the perspectives and experiences about the heatwave-health vulnerability index (HVI) tool and its impact on reducing heat-related health effects.
- Several participants have linked heatwave-health vulnerability to people living in the hottest areas.
- HVI map is perceived as a powerful tool for heatwave mitigation planning purposes and for designing heat-resilient communities.
- The participants believed that the HVI tool is currently suffering from policy attention and resourcing problems, including issues of accurately predicting heat-related health outcomes.

## INTRODUCTION

Heatwave has been recognized by the World Health Organisation (WHO) as a public health threat (WHO 2016, Beggs *et al*. 2025). Globally, seasonal heatwave-related death is estimated to be 153 073 deaths from 2009 to 2019 (Zhao *et al*. 2021). These excess deaths are expected to increase under future warming of 1.5–2°C unless effective adaptation measures are implemented (Lüthi *et al*. 2023). At the 2024 United Nations Climate Change Conference (COP29) held in Baku (Azerbaijan), nearly 200 nations agreed to invest $USD 300 billion by 2035 to develop measures to protect lives and livelihoods from extreme weather events, including heatwaves (Murphy 2024, Wei *et al*. 2025).

Australia is a high-income country with a high vulnerability to extreme temperatures, and it is estimated that frequency of days with temperatures exceeding 35°C will increase by 20%–70% by 2030, relative to 1986–2005 levels (Lawrence *et al*. 2022). Australia's future average temperature is projected to increase by 3.5°C and 2.4°C under high and medium emissions scenarios, respectively, by the end of century (Chapman *et al*. 2024). In Australia, heatwaves cause more deaths than all other natural hazards combined (Coates *et al*. 2014). Since 1844, heatwaves have killed around 5 and a half thousand people, with higher fatalities observed among older people, those with low-socioeconomic status, and disability (Coates *et al*. 2014, 2022). Epidemiological studies from Australia show that heatwave is associated with an increased risk of deaths from mental disorders (Hansen *et al*. 2008, Franklin *et al*. 2023), hospitalizations from heart attack (acute myocardial infarction) (Cheng *et al*. 2021), and increased emergency department visits from kidney disease (Xu *et al*. 2020). The risk of heat-related deaths is increasing, particularly among older people over 65 years (Cheng *et al*. 2018), children (Xu *et al*. 2014), and pregnant women (Chersich *et al*. 2020). A recent Australian study suggests that increased heatwave vulnerability is associated with heatwave-related deaths particularly among Indigenous populations (Amoatey *et al*. 2025). This is because many Indigenous/First Nations communities live in more remote areas with less housing infrastructure to mitigate/adapt to extreme heat (Kuru *et al*. 2022).

Considering the detrimental impact of extreme heat on human health, the Australian Department of Health and Aged Care released the National Health and Climate Strategy (NHCS) in 2023 aimed at addressing the impacts of climate change (e.g. extreme heat) on the health and wellbeing of Australians (DHAC 2023). The key action plan of the strategy is to develop national health vulnerability interventions, and provide guidance for risk assessment and adaptation planning to extreme heat (DHAC 2023). The heatwave-health vulnerability index (HVI) is a valuable tool that can supplement the strategy to achieve health system resilience by enhancing the population's adaptive responses to climate-related events, specifically extreme heat. HVI can be explained as the metric used to measure the degree of human vulnerability to heatwave-related health outcomes (Szagri *et al*. 2023). It is often developed from the population's sociodemographic, health, housing, and environmental data (e.g. temperature and vegetation cover) (Li *et al*. 2022). HVI is normally developed based on the Intergovernmental Panel on Climate Change (IPCC) conceptual framework of vulnerability (IPCC 2022). The IPCC defines vulnerability as a function of exposure (e.g. extreme temperature), sensitivity (e.g. elderly people with ≥ 65 years, prevalence of chronic diseases), and adaptive capacity (e.g. accessibility to air conditioning, income levels) (Buzási 2022, Szagri *et al*. 2023, Bu *et al*. 2024).

In Australia, there is growing interest in HVI studies because of increasing risk of heatwave on human health and wellbeing. HVI has been mapped across Australian cities using a wide range of temperature, socioeconomic, and health data to characterize the population's vulnerability to extreme heat (Loughnan *et al*. 2014, Wang *et al*. 2023, Li *et al*. 2024). HVI mappings are being implemented to mitigate the impact of extreme heat by various government agencies, including the New South Wales (NSW) Department of Environment and Planning (NWS 2023), the United States Centre for Disease Control (CDC) (Lehnert *et al*. 2020), and Greater London Authority (GLA 2022). However, the effectiveness of HVI in addressing extreme heat depends on how it is received, perceived, and comprehended by the relevant national/local agencies/departments who are responsible for heat management. These professionals (including clinicians, public health practitioners, emergency officers, policy advisors, meteorological officers, sociologists, etc.) are well-positioned to plan, develop, and operate HVI maps to help reduce the risk of heat-related deaths and morbidity. It is imperative to understand that technical tools like the HVI are not simply adopted or applied in a vacuum. They are interpreted, adapted, and shaped by the professionals who use them. These processes often fall outside the scope of quantitative analysis and require a more in-depth, qualitative understanding.

Although, there is a growing body of evidence about government professionals' knowledge, practices, resilience, and roles in the engagement of extreme heat management (Wilson *et al*. 2011, Brooks *et al*. 2023), there is a lack of qualitative evidence about government professional's perceptions and knowledge about the utilization of the HVI tool. The aim of this study was to qualitatively characterize the perspectives, experiences, and taught among heatwave-health management professionals (HMPs) about the need to incorporate HVI tool into heat action plans in Australia. To address this objective, the present study was conducted to answer the following research question: how do heatwave management professionals perceive and experience the HVI tool?

Regarding the contribution of this study, our findings are intended to inform climate-health planning, support institutional training, and encourage cross-sector collaboration in the implementation of heatwave vulnerability assessment tools. This will also shape future heat vulnerability research and aid in the evaluation and tracking of future heatwave mitigation and adaptation policy actions.

## METHODS

### Study design, setting, and participants

We applied a qualitative design using semi-structured interviews via Zoom with HMPs in Australia to explore their perspectives on the need to integrate the HVI tool into heat action plans. Qualitative approaches can provide a ‘wide angle picture’ through which researchers can collect rich and detailed information about how individuals experience and understand a range of issues (Toerien and Wilkinson 2004). Participants lived across the eight Australian states and territories (Queensland, New South Wales, Australian Capital Territory, Western Australia, South Australia, Tasmania, Northern Territory, and Victoria). We selected a qualitative design to seek a deeper understanding of HMPs’ knowledge, perspectives, and experiences that could inform evidence-based heatwave interventions.

### Ethics approval and data management

We obtained ethical approval from The University of Queensland's Human Research Ethics Committee (project number: 2023/HE000933). Participants’ approvals were sought, and consent was obtained to be recorded during either Zoom or Microsoft Teams interviews, and thus, to ensure anonymity, only the primary investigator (PA) will have access to the initial recorded data. Later, participants’ names and other personal details were anonymized throughout the transcript, and each participant's file was labelled (e.g. P1 for the first participant) accordingly. Finally, all the interview recorded data and related data were securely stored, accessed, and analysed on only the University's computers, which are equipped with multi-factor authentication system.

### Research team

P.A. is a final-year PhD student who has previously published on heatwave-health vulnerability project. P.A. has received training on qualitative research and ethics and conducted all the interviews, including the development of the initial coding. G.A. (PhD) and A.E.S. (PhD) have extensive experience in climate change and public health research, and they contributed to the review of codes and themes, as well as participated in thorough discussions. Senior investigators (D.P., N.J.O., Z.X.) who have served multiple roles in the field of climate and health in Australia and have engaged in several media communications. The experiences and professional backgrounds of the research team fostered the planning, design, analysis, and discussion from diverse perspectives.

### Recruitment

Participants were recruited from state departments/agencies (e.g. Health, Emergency Service, Meteorological, Climate and Environment, City Councils) responsible for the development and implementation of heat action plans (HAP) in Australia. We selected participants who met the following inclusion criteria: they should be public sector professionals working in Australia's departments whose core functions include heatwave adaptation or heatwave services. The participants were selected using both purposeful and snowball sampling strategies.

First, the research team contacted the pre-determined interviewees through emails after identifying them via Google search and social media (LinkedIn) platforms. We applied broad search terms (‘heatwave’ OR ‘heat action plans’ AND Australia OR state’) to retrieve participant's information via heat action plan (HAP) documents and other heatwave-related policy reports. We also sent an email to the administrative units of various state departments, where they assisted in identifying suitable persons for the interview. Second, both the interviewees who accepted the invitations and those who could not accept were able to refer the research team to another potential interviewee for consideration. The research team provided the description of the study and consent forms.

To increase transparency, the interview guide and ethical approval letter were also sent to participants before the interview date. Third, PA conducted online (via Zoom) in-depth semi-structured interviews between June 2024 and October 2024.

Recruitment continued concurrently with the interview phase until the research team was satisfied they had collected enough data with adequate ‘information power’ to address the research aim and question (Braun and Clarke 2021, Malterud *et al*. 2021). We considered ‘information power’ because qualitative research studies do not generally consider large sample sizes that attempt to account for the representativeness of the population. Instead, a qualitative study focuses on the depth of ideas of the study participants and the degree of detail (Braun and Clarke 2021). Therefore, this study attempted to collect enough ‘information power’ indicating that ‘the more information a sample holds, the relevant of the actual study, the lower the number of participants needed’, according to Malterud *et al*. (2016). Guided by these principles, this study intended to recruit 20 HMPs, which was deemed appropriate to achieve sufficient information from divergent perspectives about heat-health vulnerability.

### Data collection

Data collection was guided by an interview schedule (Supplemental Methods S1), which provided both direction and, flexibility, and an overall framework for the interview. The interview guide was developed by PA and reviewed by DP, AE, and ZX. At the beginning of the interview, the interviewer explained the concept of heatwave-health vulnerability to participants. The participants also shared their experiences from similar heatwave vulnerability projects they have undertaken through their respective employers (e.g. through government-funded projects and research collaborations with other institutions). These strategies enhanced the trustworthiness of the study including participants’ general understanding of the study before the main interview questions began. In view of this, we did not use any additional credibility strategies (e.g. analyst debriefs, document checks).

We applied six open-ended questions to explore participant's perceptions of the integration of HVI maps into HAP. The first question examined the knowledge of heatwave vulnerability among the participants. Then next, questions asked about the potential benefits, challenges, and recommendations for including HVI maps in HAP, and why Australia has not yet integrated (HVI) maps into HAP. The study allowed participants to discuss additional information that may be relevant to the study but was not captured by the six questions.

Participant demographic data (e.g. age, qualification, location) was also collected at the beginning of the interview. Demographic information not captured during the interview was collected via emails after the interview. All interviews were conducted in English language and lasted an average of 31.28 (18–44) minutes. The interviews were video/audio recorded using Zoom's automatic transcript function and transcribed verbatim by PA.

### Data analysis

Data analysis was conducted from 26 November 2024 to 18 February 2025. The lead author (P.A.) has previously received comprehensive training on qualitative studies and thematic analysis.

NVivo software (version 14) was used to help manage the data and analysis process. Braun and Clarke (2022)'s six-phase reflexive approach to thematic analysis was used to analyse the transcribed interview data. During phase one, PA read the transcripts repeatedly in order to become familiar with the ideas in the data (as well as anonymise any identifying information). During phase two, PA conducted sentence-by-sentence coding for interviewees’ responses that related to the aims of the study, identifying surface-level, then more latent patterns or standout ideas in the data. Phase three involved constructing themes from codes by grouping them together under common ideas. Both the codes and the themes were independently reviewed, potential discrepancies were discussed until consensus was reached by P.A., G.A., and A.E. Phase four involved revising themes to ensure they each contained distinct ideas, during which some codes were further broken down into clearer categories, or absorbed into other, better-fitting overarching themes. Phase five involved solidifying the themes by naming and briefly defining themes, followed by phase six involving the reporting of results, including through the present article (Braun and Clarke 2022). This analysis process was non-linear and involved team collaboration and discussion to debate ideas and challenge assumptions about the data.

## RESULTS

### Characteristics of the heatwave management professionals

Our study sample consisted of 20 participants from eight Australian states and territories. Half of the sample [*n* = 10 (50%)] identified as female, and three quarters [*n* = 15 (75%)] were aged 40–59 years. Queensland [*n* = 4 (20%)] was the state with the highest number of participants. A similar proportion of participants was observed from the other states and territories. Demographic information about the participants is shown in Table 1. The median professional heat-health working experience of the interviewees was 6 years (interquartile range, IQR: 2–11 years), with 10 (50%) from the state health departments, 3 (15%) each from climate/environment, and local city councils, and 2 (10%) each from meteorology, and emergency services (Table 1). The most common (20%) specialties observed among the interviewees were medicine/nursing/public health, climate policy, and environmental health/protection/wellbeing.

**Table 1.** Demographic characteristics of the study participants (*n* = 20) represented as sample (*n*) and percentage (%).

Participants	
**(A) Sex**	
Male	10 (50%)
Female	10(50%)
**(B) Age (years)**	
20–39	4 (20%)
40–59	15 (75%)
60–79	1 (5%)
**(C) Highest educational qualification**	
PhD	3 (15%)
Masters	3 (15%)
Bachelors	6 (30%)
Diploma/certificate^a^	8 (40%)
**(D) States**	
Australian capital territory	3 (15%)
Queensland	4 (20%)
New South Wales	2 (10%)
Victoria	2 (10%)
South Australia	3 (15%)
Western Australia	2 (10%)
Northern Territory	3 (15%)
Tasmania	1 (5%)
**(E) Professional work experience (years), median (IQR)**	6 (2–11)^b^
**(F) Speciality/field**	
Medicine/nursing/public health	4 (20%)
Climate policy	4 (20%)
Disaster/emergency services	3 (15%)
Environmental health/protection/wellbeing	4 (20%)
Climate/atmospherics science/environment	5 (25%)
**(G) Departments/agencies**	
Health	10 (50%)
Climate/environment	3 (15%)
Emergency services	2 (10%)
Meteorology	2 (10%)
City council	3 (15%)

^a^Including graduate diploma, graduate certificate, Associate Diploma, Diploma, and High School Certificate. ^b^Refers to median [interquartile range (IQR)]. Note: The median (IQR) duration (minutes) of the interview is 32 (27–35).

### The overarching interview themes and subthemes

**Figure 1.**
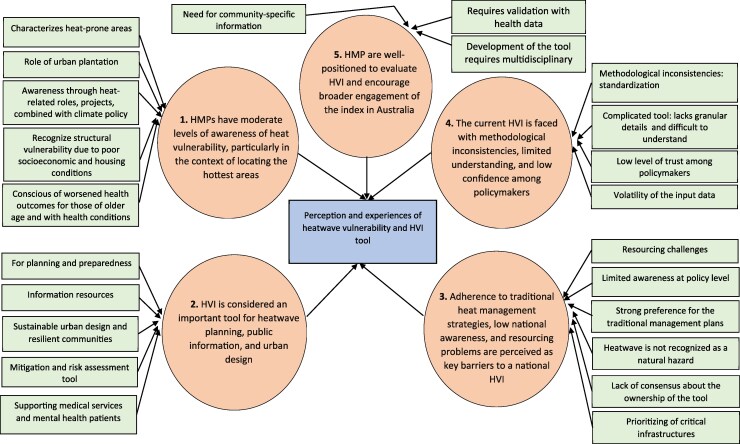
Graphical synthesis of the themes and subthemes. The arrows indicate how the subthemes relate to the themes, and how the themes relate to the overall study aim (in rectangular box).

Five major themes were constructed: (i) HMP have moderate levels of awareness of heat vulnerability, particularly in the context of locating the hottest areas, (ii) HVI is considered an important tool for heatwave planning, public information, and urban design, (iii) adherence to traditional heat management strategies, low national awareness and resourcing problems are perceived as key barriers to a national HVI, (iv) the current HVI is faced with methodological inconsistencies, limited understanding, and low confidence among policymakers, and (v) HMP are well-positioned to evaluate HVI, and encourage broader engagement of the index in Australia. Supplementary Tables S1–S5 contain the list of the main themes, subthemes, and key illustrative quotes, and the Fig. 1 presents a graphical synthesis of the themes and subthemes.

#### Theme one: HMP have moderate levels of awareness of heat vulnerability, particularly in the context of locating hottest areas

##### Characterize heat-prone areas

Heatwave-health management professionals (HMPs) understood heat vulnerability by linking it to the cities with high surface temperatures. Heat vulnerability was seen as a climate change issue that is modulated by land use features (e.g. infrastructures, greenery). HMPs stressed that living in hotter cities with poor outdoor cooling systems and heat absorbing structures exacerbate the risk of heat vulnerability. A heatwave professional elaborated:

‘…So, people were having lots of concrete around them, a real concrete jungle, and they didn't have air conditioning built in, so people were relying on fans, so they were considered a lot more vulnerable….’ (Participant 18, Female, SA, Health).

##### Role of urban plantation

Regardless of the level of heatwave vulnerability, HMPs noted that having access to cool environments and shade in the hottest areas within the cities could reduce the adverse effect of heat on people's health. These areas can be established by accurately identifying high-temperature hotspots, especially in residential areas and streets, and encouraging tree planting programmes, and creating more parks. Two HMPs from South Australia (SA) and New South Wales (NSW) elaborated that:

‘I guess, and there are parts of public health that are involved with greener spaces and initiatives within South Australia to promote better environments that reduce heat’ (Participant 14, Male, SA, Health).and ‘I also had previously led a program of work characterizing the urban heat island and heat vulnerability in Sydney, where we looked and tracked land at the tree cover and canopy cover’ (Participant 20, Male, NSW, Climate/Environment).

##### Awareness through heat-related roles, projects combined with climate policy

HMPs did not generally receive any formal education and training on heat health but became acquainted with heat vulnerability as part of their functional job responsibilities and through state-wide and jurisdictional climate adaptation policies. Their involvement in state-funded heat intervention projects and participation in high-level climate policy meetings were two important sources of heat vulnerability knowledge for HMPs. A HMP from Queensland State (QLD) Emergency Services department elaborated, ‘we built a vulnerability index as part of an ongoing component of that project, and that looks at various kinds of adaptive capacities and coping capacities that exist within communities’(Participant 16, Male, QLD, Emergency Services). ‘It's really sort of popped up at a national level through the National Climate Change Adaptation Strategy, and I think that the Commonwealth, Department of Health and Aging is starting to do a lot more work around heat-health and adaptation’ (Participant 01, Female, NT, Health).

##### Recognize structural vulnerability due to poor socioeconomic and housing conditions

HMPs understood that key social determinants of health have contributed to heatwave vulnerability, including income instability, unemployment, low education, housing instability, and minority status. Several HMPs iterated that improving livelihood and wellbeing could reduce vulnerability. One HMP said: ‘…look at a number of different social factors and economic factors I guess around areas within South Australia, look at anything from housing stock and their ability to cope with heat and heat vulnerabilities through to economic impacts on people being able to make improvements to their living situations and circumstances’ (Participant 14, Male, SA, Health). Similarly, being homeless because of limited accessibility to social benefits can explain vulnerability to heatwaves among specific disadvantaged communities. One HMP elaborated that ‘I was saying, before, we have a lot of people come in from remote communities and they will just sleep rough in parks, and that's different, and so we might include that information. These are the typical areas that they are, and they don't have anything to do with the social housing’ (Participant 13, Female, NT, City Council).

##### Conscious of worsened health outcomes for those of older age and with health conditions

HMPs shared how certain unhealthy living conditions such as being sick, having chronic diseases, and aging make individuals more vulnerable to heatwave-related health complications. One HMP elaborated: ‘So, we know that there's going to be comorbidity, there's going to be high levels of comorbidity, there's going to be high elderly population, so presumably more vulnerable to heatwave events’

(Participant 15, Female, QLD, Health). A female HMP mentioned that mental health patients are vulnerable to heatwave and therefore require support. ‘So, you know, some people are really quite unwell, and don't have any concept of whether it's hot or cold outside, they will dress in 16 layers regardless of the temperature’ (Participant 18, Female, SA, Health).

#### Theme two: HVI is considered an important tool for heatwave planning, public information, and urban design

##### For planning and preparedness

HMPs have acknowledged the HVI map as an essential tool for heatwave management. However, the tool is more suitable for planning and preparedness for an imminent heatwave event. An HMP from the Emergency Service Department highlighted that an HVI can assist in prioritizing vulnerable areas and inform the type of heatwave responses that needs to apply to that area. ‘…it'd help with planning, essentially like you wanna you want to have a list of actions, a list of the risks and vulnerabilities within your community…’ (Participant 16, Male, QLD, Emergency Services). This planning strategy, overtime, will contribute to building long-term extreme heat-related adaptation strategies among communities. An HMP from health department elaborated: ‘…knowing about where heat vulnerability is, can help us more in that preparedness, planning phases, you know, and sort of building that community resilience to heatwave’ (Participant 01, Female, NT, Health).

##### Information resources

Providing heatwave-related knowledge and intelligence was another important core function of the HVI map, as raised by the HMPs. The tool was seen by them as an educational resource that can inform the public and influence the policy makers understanding of extreme heat as an important climate hazard. HMPs elaborated that: ‘I think things like heat vulnerability maps are an important communication tool to raise the profile of heat as a hazard of critical importance that might focus the minds of governments on heat as a hazard’ (Participant 04, Male, QLD, Climate/Environment), and ‘To be honest, I think it's probably a better education and engagement piece for broader stakeholders, and whether that's community groups, non-governmental organisations (NGOs), even like community members as well to kind of get a sense of the vulnerability within their area' (Participant 15, Female, QLD, Health).

##### Sustainable urban design and resilient communities

HMPs believed that the importance of the HVI tool is not limited to only knowing the levels of heatwave-related risks at the population level but could also support the overall design and planning of smart cities that can withstand extreme heat. The HMPs said that the tool: ‘…helps with our urban design planning’ (Participant 17, Female, VIC, City Council), and ‘…sort of building that community resilience to heatwave’ (Participant 01, Female, NT, Health).

##### Mitigation and risk assessment tool

Communities could benefit from the HVI map because it can assist in locating populations that are likely to suffer from health problems prior to heatwave events. The tool could also be incorporated into environmental or health impact assessment framework to help protect communities against heatwaves. This point was raised by an HMP from department of health. Some HMPs assert that the HVI map can help implement heatwave mitigation strategies, such as the installation of cooling systems in city hot spots and tree planting, but it will depend on the socioeconomic status of the communities. Highly vulnerable or poor communities can benefit from cooling systems such as air conditioning and improved housing designs to withstand extreme heat. This view was shared by one HMP from emergency services department.

##### Supporting medical services and mental health patients

Assisting in effective healthcare delivery was an important benefit of the HVI map cited by HMPs as it makes it easier for clinicians to understand the underlying environmental-related causes in the spark in health outcomes during heatwave events. A female HMP mentioned that the tool is important for mental health management. This was elaborated as: ‘…there's an expectation that every person in mental health will have this vulnerability assessment tool completed on them and then have a determination about whether they're actually at risk in an extreme heat episode…’ (Participant 18, Female, SA, Health).

#### Theme three: adherence to traditional heat management strategies, low national awareness and resourcing problems are perceived as key barriers to a national HVI

##### Resourcing challenges

There were varied perceptions among the HMPs with regards to the range of resource issues affecting the implementation of the HVI tool. For some, the majority of the local councils within the state do not have the financial capacity to develop and operate HVI map. Other HMPs feel that there is limited technical expertise in their state to be able to independently implement the HVI map. An alternative approach is to learn and gain experience about how the tool is being used by other States due to the lack of these technical resources.

##### Limited awareness at policy level

Heatwave management policies usually require government review and approval. On the contrary, HMPs perceive that the HVI tool has not received the needed attention it deserves either at federal, state or jurisdiction government level. A broader perception was shared among several HMPs that there is a lack of overall heatwave and related mitigation policies at the government level. A Western Australian HMPs shared ‘I think that Australia, is that a fairly low level of urban heat or heat generally awareness. So well, speaking for WA, I think a couple of years ago a paper I saw, WA became last on a list of the mainland States in terms of heat, urban heat island (UHI) awareness and UHI mitigation policies’ (Participant 05, Male, WA, Climate/Environment).

##### Strong preference for the traditional management plans

HMPs prefer to continue to depend on the local weather forecasting systems for heatwave management because they are easier to use than the HVI tool. This point was elaborated as: ‘…the vulnerability mapping from our perspective, we are aware of it but we're more driven around the warning systems’ (Participant 14, Male, SA, Health). The professionals iterated that the traditional weather systems are more effective in assessing heatwave conditions at local level.

##### Heatwave not recognized as a natural hazard

Heatwaves were seen as emerging emergencies and undramatic events, and they rarely caused the public to become overwhelmed. HMPs elaborated, ‘…emergency management agencies don't recognize heat as a natural disaster, or emergency event…’ (Participant 04, Male, QLD, Climate/Environment). Other HMPs believe that unlike other disasters such as fire which is destructive and may echo in the minds of people for longtime while heatwave does not, quickly disappears within space. A female HMP elaborated that: ‘So yeah, I just think it's because we had those really huge fire events in 2019 and 2020 that they were in the forefront of everyone's mind, and the impact was dramatic, and all the other hazards might have disappeared a little bit behind it. Yeah, but heatwave is not there, If that makes sense’ (Participant 12, Female, ACT, Meteorology).

##### Lack of consensus about the ownership of the tool

HMPs shared common views about ownership issues of the HVI tool. For some HMPs, there seems to be tension among the State agencies and departments about who should manage the HVI map. Other HMPs shared that the tool lies at the intersection between emergency, meteorological, and health services. HMPs elaborated, ‘…who's gonna own the system, who's gonna host it, where's it live, because again, while we are the hazard management agency, community vulnerability is not something that the Health Department suppose to owns. If that's why put it so, you know. Is that more of a State government? Or does it sit in a department of communities or a community sort of space?’ (Participant 06, Male, WA, Health).

##### Prioritizing of critical infrastructures

Overall, the interviewed HMPs confirmed that it is difficult to implement the HVI map because of government interest in protecting major infrastructures from heatwave impacts rather than human health. HMP elaborated, ‘So, there's a lot more focus on those infrastructure damage, basically and rather than events that lead to human health impact…’ (Participant 04, Male, QLD, Climate/Environment). Other HMPs shared the view that there is no special focus on human health, but rather, a broader government policy framework on protecting railways, and electrical power systems including human health.

#### Theme four: the current HVI is faced with methodological inconsistencies, limited understanding, and low confidence among policymakers

##### Methodological inconsistencies: standardization

Not having a clear-cut standard approach to developing the HVI map was cited by the HMPs as one of the reasons affecting the development and operation of the national HVI map. They saw inconsistencies as HVI maps coming from different sources and using different datasets, making it difficult for them to make informed decisions about the appropriate HVI map. An HMP shared that ‘…there's a risk that there'll be these kinds of products coming from multiple directions using different data sets, different versions of metrics from various places that could get a bit messy and difficult to navigate across the board’ (Participant 04, Male, QLD, Climate/Environment). Some HMPs have seen this as a lack of coordination among State agencies and academic institutions during the development of the map, making it difficult to achieve standardization.

##### Complicated tool: lacks granular details and is difficult to understand

The HMP's views about the complexity of the HVI tool varied. A Victoria-based HMP elaborated, ‘So, you've sort of run the risk of providing it an interpretation of the data, so what does it mean? I think it's really the hardest part’ (Participant 08, Female, VIC, Emergency Services). To some, lack of clear interpretation and limited heat vulnerability-related detailed information among communities hindered the recognition of the tool at the national level. Other HMPs shared that, at times, people with technical knowledge are required to understand the interpretation of the HVI map.

##### Low level of trust among policymakers

The positive attitude towards the HVI tool is a key factor determining its acceptance by the policymakers. HVI tool was seen as not having yet achieved strong confidence and trust among relevant government agencies/departments for the purpose of heatwave management. Two male HMPs shared that: ‘…policymakers are not confident in how they should use the data’ (Participant 03, Male, SA, Meteorology) and ‘…if you expect a spike …in ambulance turnouts or hospitalizations are your observations matching what you expected to happen? I think, until you can kind of test it in theory, it's very difficult to have confidence in a model’ (Participant 16, Male, QLD, Emergency Services).

##### Volatility of the input data

Uncertainties about rapid changes in HVI status among communities due to changes in sociodemographic characteristics, and the concern about reliance on historical data for the HVI map were the major factors hindering the implementation of national HVI map.

HMPs still believe that heat vulnerability, or HVI, has volatility issues and could change over time in response to demographic shifts and infrastructural development. Two HMPs elaborated these points as: ‘Once you get into a vulnerability map, it's got all those built social natural factors in there that are also going to change over time, so it just makes it very challenging from the climate longer term climate perspective’ (Participant 04, Male, QLD, Climate/Environment), and ‘What I think is that the heat vulnerability indices are going to change rapidly as infrastructure changes, as populations change, as immigrants arrive. So, it's something that needs to be able to be dynamically refreshed on a fairly rapid update as an asset so that planners can see where risk is growing and diminishing across communities, whether by location or by type of community’ (Participant 03, Male, SA, Meteorology).

#### Theme five: HMP are well-positioned to evaluate HVI, and encourage broader engagement of the index in Australia

##### Requires validation with health data

The HVI tool's ability to realistically predict death cases and morbidity during heatwave events was the main requirement recommended by the HMPs, making it easier for policymakers to accept and implement the tool at the national level. HMP from Climate/Environment Department elaborated, ‘You look at the data, including hospital admissions just to see what's happening is there evidence that the index that you've developed does it track? High vulnerability equal higher number of admissions, that sort of thing’ (Participant 05, Male, WA, Climate/Environment). Positive evaluation outcomes of the HVI help bring confidence and certainty when using it to assess heat-related health outcomes among communities.

##### Development of the tool requires multidisciplinary engagement

The interviewed HMPs shared a holistic view that involving professionals of different specialties within government, academics, and other relevant stakeholders in the development of the HVI tool will ensure national standardization and how to operate the tool. A HMP from South Australia (SA) elaborated, ‘You need to bring together the communities that are actually engaged in generating the indices and the responses to those indices. To make sure that people have confidence, and how they should use them’ (Participant 03, Male, SA, Meteorology).

##### Need for community-specific information

HVI was seen by the HMPs as a community-specific tool whose development should be tailored to meet the needs of the local communities. Some HMPs from Northern Territory (NT) elaborated, ‘I think one of the reasons is type of people who are the vulnerable people might differ in each city. So, the vulnerable people in Darwin are very different to, say, homeless people in Sydney or Melbourne they got completely different, like the typical circumstances that they're in are just so different to the type of vulnerable people in Darwin, and I don't think you can use the same criteria’ (Participant 13, Female, NT, City Council), ‘So, if for instance, somebody is developing a heat vulnerability index they will have to look at these specific characteristics of each community and take that into consideration, If you lump up everything, it wouldn't be useful' (Participant 02, Male, NT, Health).

## DISCUSSION

This current study investigates the perceptions and experiences of HMPs on the HVI assessment tool in Australia. This study has improved our qualitative understanding of HVI mapping in the context of heat-health planning, to our knowledge that the question of HVI perception has been studied using a qualitative interview approach and covering a diverse range of HMPs. The study found limited contrasting views on how HMPs perceive and experience HVI, including in relation to heatwave vulnerability in the context of the hottest areas within cities, the role of urban vegetation, and the exacerbated impacts for individuals with low socioeconomic status, poor health conditions (e.g. chronic diseases, patients), and poor housing conditions.

Some HMPs become aware of HVI by taking part in local heatwave-related projects and familiarizing themselves with Australian national climate policies. However, HMPs understood heat vulnerability as being independently attributed to the above risk factors but not based on the conceptual framework of vulnerability, which is the degree of exposure (e.g. living in hot areas), sensitivity (e.g. being an older person), and adaptive capacity (e.g. housing conditions; Adnan *et al*. 2022, Côté *et al*. 2024).

Our findings were consistent with earlier works that found that Australian professional's knowledge of heat management was based on climate-related planning, education, and the use of heat-mitigating structures such as cooling systems and tree plantations (Smith *et al*. 2024). Other past studies mentioned that most stakeholders believe that increasing rental houses without air conditioning, low-income earnings, and the burden of mental health conditions are major factors linked to heatwave vulnerability (Hansen *et al*. 2014). Several HMPs, particularly from health sectors, assert that the importance of the HVI tool is not only for general planning and preparedness for imminent heatwave events but can assist in sustainable urban design, building resilient communities, and overall risk assessment. Similar to this current work, a study in the United States involving over 90 jurisdictions showed that both heat-related risk communication and surveillance systems are important for heatwave response (Errett *et al*. 2023). The main difference between the surveillance systems and HVI maps is that the former involves real-time monitoring and forecasting of heatwave vulnerability. Another qualitative study conducted in the state of Queensland, Australia perceived a similar HVI-related tool (early warning system) as an effective tool for enhancing heat-related resilience among older adults and issuing cooling advice (Oberai *et al*. 2025). Although, this tool assesses risk and supports mitigation (alerting the vulnerable people and providing advice for cooling), it is still an emerging tool and has not yet been used for practice.

Some of the HMPs shared a common view that the HVI map has not yet been used for heatwave management in Australia because of resourcing challenges, limited awareness at the policy level, and preference for traditional weather (heatwave) forecasting plans. Few of them hinted that there is a lack of interest in using HVI because heatwaves, in general, have not been recognized as a natural hazard in Australia. Recognition of extreme heat as an emergency issue has created tension among Australian institutions, as reported by past studies (Bolitho and Miller 2017). The interest has focused on the impact of extreme heat on electrical power supplies and major critical infrastructure without addressing heat as a health-related chronic stress factor. On the contrary, HMPs from the United States consider heatwave as a threat to public health and wildlife. They are of the view that the plans to mitigate it have been hindered by financial resources and politics (Meerow and Keith 2022). This underscores the need for governments to provide urgent financing for heatwave intervention projects, with good coordination between hospitals and public health agencies. The government can also recognize extreme heat mitigation policies under the United Nations Sustainable Development Goals, particularly under Goal 3 (global health and well-being) and Goal 13 (climate action) to help attract funding and international interest (United Nations 2015).

The awareness of heat-health vulnerability among the HMPs will encourage sectoral training and collaboration, particularly in the development, interpretation, and operation of HVI. Almost all the HMPs agree that HVI can support long-term heatwave-resilient programming, prioritization of adaptation programs, and mitigation of risk through preparedness as well as planning.

For example, integrating the HVI tool into the existing heat-health action plans (HHAPs) for public education and public health messaging prior to extreme heat events could improve the reduction of heat-related deaths and morbidity cases, such as hospitalizations and emergency department visits (Osborne *et al*. 2024). This is because the implementation of only the HHAPs for the past 10 years in Australia has not achieved any significant reduction in heat-related health. Post HHAPs implementation across an Australian state (Victoria) from 2010 to 2019 led to only 3.4% reduction in heat-related deaths (Osborne *et al*. 2024).

In Australian cities, the post-HHAP implementation (2010–2019) resulted in a 0.12% reduction in heat-related deaths among the population. This reduction may be attributed to the quality of healthcare services and housing systems (e.g. accessibility to AC, good insulation systems) in urban areas compared to non-urban communities, such as remote/rural parts of Australia. Thus, integrating HVI into HHAPs during the planning stage of heatwave mitigation will help identify the most vulnerable populations for tailored heatwave adaptation intervention strategies, leading to a significant reduction in heat-related health risks following the implementation of HVI-HHAPs.

Regarding potential challenges of the current HVI tool, it emerged that some HMPs were concerned with methodological inconsistencies such as a lack of standardization and a low level of trust among policymakers. However, several HMPs recommended the evaluation of the HVI tool with observational health outcome data. Previous interview studies conducted in India have questioned the methodological issues regarding who is considered the most vulnerable to heat (Singh *et al*. 2024). It was emphasized that current heat vulnerability assessment tools hardly account for mobile populations such as outdoor workers, including pregnant/lactating mothers, and their related socioeconomic disparities (Singh *et al*. 2024). HMPs have advised that the HVI tool should be tested if it can predict deaths and morbidity such as increased in hospitalizations above the baseline during heatwave events. However, most HVI validation processes utilize only historical health data, making it difficult to predict future heat-related outcomes. In addition, the prediction accuracy of some HVI models is only 54% and thus cannot account for the remaining proportion of heat-related health outcomes (Bao *et al*. 2015). More comprehensive studies are urgently required to address these discrepancies.

## LIMITATIONS

We identified a number of limitations in this study. First, all perspectives among the HMPs were not captured as each is shaped by individual experience and creation of meaning. Thus, early career HMPs with few years of experience may have had limited time to fully understand the complexities of HVI tools. However, they may offer more novel or innovative perspectives. Future studies might seek the perspectives of more experienced HMPs. Second, HMPs living in colder regions of Australia may have different perspectives on vulnerability due to low frequency of heatwave events in their areas. Fourth, institutional information-sharing privacy policies may have influenced their interview responses. For example, HMPs involved in on-going institutional HVI project may not be able to share the detail perspectives about the tool due to their institutional data confidentiality policies. Fifth, the thematic analysis approach, code/theme development, can be influenced by the researchers’ knowledge or ideology on the topic, potentially distorting the actual study outcomes. To overcome this, researchers must ensure adequate reflexivity by remaining close to transcripts during code/themes development, as well as effective team discussions and brainstorming (Roberts *et al*. 2019).

## CONCLUSION

This qualitative study explored HMPs’ perceptions and experiences of heatwave vulnerability and the HVI tool. HMPs found several benefits of the HVI tool, including the associated challenges, and recommended strategies to improve its practical application. HMPs’ understanding of heat vulnerability as areas representing the hottest parts of cities, the benefits of HVI for planning/preparedness, the inherent resourcing challenges, limited awareness at the policy level, and the need for evaluation with health outcome data were the main determinants of HVI perception. There is a need to implement the recommendations suggested by HMPs into future HVI tools.

## Supplementary Material

daaf221_Supplementary_Data

## Data Availability

The data cannot be made publicly because they contain sensitive personal information.
