# Supplementary material for: A qualitative study on utilization of vulnerability assessment tool for heatwave-related health adaptation intervention in Australia
Source: Health Promot Int. 2025 Dec 19;40(6):daaf221. doi: 10.1093/heapro/daaf221 (PMC12715503; doi:10.1093/heapro/daaf221)
Supplement: daaf221_Supplementary_Data [file daaf221_supplementary_data.docx]

**A qualitative study on utilization of vulnerability assessment tool for heatwave-related health adaptation intervention in Australia**

Patrick Amoatey^1*^, Grace Arnot^2^, Aklilu Endalamaw^1^, Nicholas J.Osborne^1,3,4^, Zhiwei Xu^5^, Dung Phung^1^

^1^School of Public Health, Faculty of Health, Medicine and Behavioural Sciences (HMBS), The University of Queensland, 288 Herston Road, Herston, QLD 4006, Australia

^2^Institute for Health Transformation, Faculty of Health, Deakin University, 1 Geringhap Street, Geelong, VIC 3220, Australia

^3^School of Population Health, University of New South Wales, 55 Botany Street, Randwick, NSW 2031, Australia

^4^European Centre for Environment and Human Health (ECEHH), University of Exeter Medical School, Peter Lanyon Building 12, Penryn, Cornwall, TR10 8RD, United Kingdom

^5^School of Medicine and Dentistry, Griffith University, Parklands Drive, Southport, Gold Coast, QLD 4222, Australia

*Corresponding author: School of Public Health, Faculty of Health, Medicine and Behavioural Sciences (HMBS), The University of Queensland, 288 Herston Road, Herston, QLD 4006, Australia, E-mail: [olando2009@yahoo.com](mailto:olando2009@yahoo.com)

**Supplemental Methods S1: Interview guide**

**Study Aim:** To investigate the perception and experience of heatwave vulnerability maps among heatwave management professionals across Australia using a qualitative interview approach.

**Specific objectives:**

1. To understand the perception of the heat vulnerability assessment index (HVI) among the key climate change and health stakeholders.

2. To explore the potential benefits, challenges, and future recommendations of integrating HVI mapping into future current heat action plans.

**Introduction**

- Introducing myself to participants.
- Explain the aim of the study.
- Stating the duration of the interview.
- Explaining that the responses are confidential and de-identified. Respondents may decide to decline to answer certain questions or may refuse to participate in the interview or discontinue the interview at any point during the interview.
- Ensuring that participants have read the information sheet online.
- Ensuring that participants have accepted the consent form.

**The Key Areas of the Interview**

- Perception of heat vulnerability index maps.
- Benefits of integrating heat vulnerability index maps into Australia's heat action plans.
- Potential challenges of integrating heat vulnerability index maps into heat action plans.
- How to Improve the effectiveness of heat vulnerability index maps into heat action plans in the future.

**(A) Part one:**

*Participant’s Demographic and Professional Information.*

- Age
- Sex (Male or Female)
- Highest Educational level
- Citizenship status (Australian citizen , Not Australian Citizen)
- Years living in Australia
- State you are living
- Name of your institution/department
- Your position
- Your role in the department
- Number of years spent working on heat-health/climate-health or your role

**(B) Part two:**

*(I) Knowledge of the Vulnerability Index Map*

1. **Are you aware of the heat vulnerability index map?**

Interviewer’s Probes:

- Can you share where you came across it?
- Does Australia have one at a national level?
- What about the States?

*(II) Integration of Vulnerability Index Map in Heat Health Plans*

**(2) Do you think it is important to integrate the heat vulnerability index map into State-level heat health plans or State Emergency Management Plan Extreme Heat Sub-Plan?**

**in Australia?**

Interviewer’s Probes:

- Can the map better identify highly or least vulnerable areas?
- Can the map help develop targeted interventions that meet the needs of vulnerable communities?
- What specific community-based interventions can the map help to develop?
- Can the map effectively mitigate heatwaves compared to current heat public messaging heat alert systems? -Can you share some examples?
- Can the map improve the effectiveness of the current heat action plans when integrated?

**(3) Would you recommend the integration of the heat vulnerability index map into State-level heat health plans or State Emergency Management Plan Extreme Heat Sub-Plan?**

**in Australia?**

Interviewer’s Probes:

- Can you elaborate on any specific reasons?
- Would it help improve the effectiveness of the current heat action plans?-How?

**(4) Do you have any idea why Australia has not yet integrated a heat vulnerability index map into State-level heat health plans or State Emergency Management Plan Extreme Heat Sub-Plan?**

*(III) Potential Challenges and Recommendation of Vulnerability Index Map*

**(5) What are the potential challenges that could be associated with the integration of vulnerability index maps into State-level heat health plans or State Emergency Management Plan Extreme Heat Sub-Plan in Australia?**

**(6) Do you have any recommendation about the integration of vulnerability index maps into State-level heat health plans or State Emergency Management Plan Extreme Heat Sub-Plan in Australia?**

Interviewer’s Probes:

- Could that be reliability issues?-Challenges of identity heat-related deaths and morbidity?
- Could that be a methodological issue?- Difficulties of identifying the appropriate indicators-Lacking appropriate statistical approach - Does not determine causal effects?
- Could that be implementation challenges?-Resource intensive,-Capital intensive?
- Public perception issues?-Can the use of a heat vulnerability index map deter people from living in areas identified with high heat vulnerability?-Can the use heat vulnerability index affect housing sales/rent that have high heat vulnerability?

**Before ending the interview, is there anything you would like to add to this topic ?**

Thank you so much for participating in these in-depth interviews, I appreciate your time.

**-------------------------------------------------------------------------------------------------------------------------**

**END OF INTERVIEW**

**Table S1.** Theme one: HMPs have moderate levels of awareness of heat vulnerability, particularly in the context of locating the hottest areas

| **Subthemes** | **Supporting quotes (participant)** |
| --- | --- |
| Characterizes heat-prone areas | “…So, people were having lots of concrete around them, a real concrete jungle, and they didn't have air conditioning built in so people were relying on fans, so they were considered a lot more vulnerable….” Participant 18, Female, SA, Health |
|  | “I understand there was some heat mapping undertaken, and it was introduced and used in some of the original plans and some research that was done around heat sinking cities and things like that ” Participant 08, Female, VIC, Emergency Services. |
| Role of urban plantation | “I also had previously led a program of work characterizing the urban heat island and heat vulnerability in Sydney, where we looked and tracked land at the tree cover and canopy cover” Participant 20, Male, NSW, Climate/Environment |
|  | “I guess, and there's parts of public health that are involved with greener spaces and initiatives within South Australia to promote better environments that reduce heat” Participant 14, Male, SA, Health |
| Awareness through heat-related roles, projects, combined with climate policy | “We built a vulnerability index as part of an ongoing component of that project, and that looks at various kind of adaptive capacities and coping capacities that exist within communities”,  Participant 16, Male, QLD, Emergency Services |
|  | “It's really sort of popped up at a national level through the National Climate Change Adaptation Strategy, and I think that the Commonwealth, Department of Health and Aging is starting to do a lot more work around heat-health and adaptation” Participant 01, Female, NT, Health |
| Recognise structural vulnerability due to poor socioeconomic and housing conditions | “……look at a number of different social factors and economic factors I guess around areas within South Australia, look at anything from housing stock and their ability to cope with heat and heat vulnerabilities through to economic impacts on people being able to make improvements to their living situations and circumstances” Participant 14, Male, SA, Health. |
|  | “I was saying, before, we have a lot of people come in from remote communities and they will just sleep rough in parks, and that's different, and so we might include that information. These are the typical areas that they are, and they don't have anything to do with the social housing” Participant 13, Female, NT, City Council. |
| Conscious of worsened health outcomes for those of older age and with health conditions | “So, we know that there's going to be comorbidity, there's going to be high levels of comorbidity, there's going to be high elderly population, so presumably more vulnerable to heatwave events”  Participant 15, Female, QLD, Health |
|  | “So, you know, some people are really quite unwell, and don't have any concept of whether it's hot or cold outside, they will dress in 16 layers regardless of the temperature” Participant 18, Female, SA, Health |
|  | “…..that might be say, pregnant moms as an example, or even if you just come out of hospital, obviously you are compromised in some way” Participant 06, Male, WA, Health |

**Table S2.** Theme two: HVI is considered an important tool for heatwave planning, public information, and urban design

| **Subthemes** | **Supporting quotes (participant)** |
| --- | --- |
| For planning and preparedness | “So, you it'd help with planning, essentially like you wanna you want to have a list of actions, a list of the risks and vulnerabilities within your community before an event happens”  Participant 16, Male, QLD, Emergency Services |
|  | “But it's sort of like I said our heatwave management plan has been about responding to heatwave, whereas I think the you know, knowing about where heat vulnerability is, can help us more in that preparedness, planning phases, you know, and sort of building that community resilience to heatwave” Participant 01, Female, NT, Health |
| Information resources | “I think things like heat vulnerability maps are an important communication tool to raise the profile of heat as a hazard of critical importance that might focus the minds of governments on heat as a hazard” Participant 04, Male, QLD, Climate/Environment |
|  | “To be honest, I think it's probably a better education and engagement piece for broader stakeholders, and whether that's community groups, non-governmental organisations (NGOs), even like community members as well to kind of get a sense of the vulnerability within their area” Participant 15, Female, QLD, Health |
| Sustainable urban design and resilient communities | But, as I said, it's just not a tool that our own Department of Health has really, I suppose, used as a tool to help us, but we're aware of it. I think our climate change teams are probably you know they started it, they need it and helps with our urban design planning. Participant 17, Female, VIC, City Council |
|  | “….knowing about where heat vulnerability is, can help us more in that preparedness, planning phases, you know, and sort of building that community resilience to heatwave**”** Participant 01, Female, NT, Health |
| Mitigation and risk assessment tool | "We could consider measures particularly for those higher vulnerable areas. I'd be willing to see the map. Then I think also, we could potentially incorporate it into our risk assessment kind of protocol, which is where we kind of look at what the area the population affected will be by the heatwave" Participant 11, Female, TAS, Health |
|  | “……advice for an affluent community that has really good access to well-constructed buildings and insulated and air-conditioned buildings, the advice is going to be very different to a community that might be more rural or regional, that doesn't have access to those amenities” Participant 16, Male, QLD, Emergency Services. |
| Supporting medical services and mental health patients | “For instance, if the health sector was seeing an increase in requests for ambulances or hospital admissions, it means that they could, prepare and plan for that leading up to an event rather than being reactive” Participant 16, Male, QLD, Emergency Services |
|  | “So, we've put that heat vulnerability assessment tool into service, and there's an expectation that every person in mental health will have this vulnerability assessment tool completed on them, and then have a determination about whether they're actually at risk in an extreme heat episode or if they have got mitigating strategies that make them less vulnerable ” Participant 18, Female, SA, Health. |

**Table S3.** Theme three: Adherence to traditional heat management strategies, low national awareness, and resourcing problems are perceived as key barriers to a national HVI

| **Subthemes** | **Supporting quotes (participant)** |
| --- | --- |
| Resourcing challenges | "Unfortunately, that's council dependent and resource dependent, that is something that I've tried to raise in terms of what would need to be done at a state level funding for councils because there's 77 councils in Queensland, and the majority of those can't afford to do that work " Participant 15, Female, QLD, Health. |
|  | “We don't necessarily have the resources to kind of pick up these things and lead ourselves, I'd be interested to know if other, how other jurisdictions are using it, but one thing we are doing differently for the 2024 -2025 summer period is we have a heatwave strategy” Participant 11, Female, TAS, Health. |
| Limited awareness at policy level | “Yeah, I suppose this is just my personal thoughts, because I don't feel like I've ever heard the heat vulnerability map being discussed broadly within government” Participant 09, Male, NSW, Health. |
|  | “I think that Australia, is that a fairly low level of urban heat or heat generally awareness. So well, speaking for WA, I think a couple of years ago a paper I saw, WA became last on a list of the mainland States in terms of heat, urban heat island (UHI) awareness and UHI mitigation policies” Participant 05, Male, WA, Climate/Environment. |
| Strong preference for the traditional management plans | “There have been protocols in place for over 5 years but the way it has been done traditionally is very much based on a local assessment following alert that there will be a heatwave from the BOM” Participant 11, Female, TAS, Health |
|  | “……the vulnerability mapping from our perspective, we are aware of it but we're more driven around the warning systems” Participant 14, Male, SA, Health |
| Heatwave is not recognized as a natural hazard | “A big thing, I think, when it comes to the disaster management side is that a lot of emergency management agencies don't recognize heat as a natural disaster, or emergency event, it doesn't come under the national disaster arrangements, so it goes under the radar a little bit” Participant 04, Male, QLD, Climate/Environment |
|  | “So yeah, I just think it's because we had those really huge fire events in 2019 and 2020 that they were in the forefront of everyone's mind, and the impact was dramatic, and all the other hazards might have disappeared a little bit behind it. Yeah, but heatwave is not there, If that makes sense” Participant 12, Female, ACT, Meteorology |
| Lack of consensus about the ownership of the tool | “It's always been a tension between heatwave and emergency services and the health agencies as well who takes responsibility for it, I think over the last couple of years It's evolved that all agencies need to step up given It's one of our biggest risks in terms of hazards” Participant 12, Female, ACT, Meteorology |
|  | “…… who's gonna own the system, who's gonna host it, where's it live, because again, while we are the hazard management agency, community vulnerability is not something that the Health Department suppose to owns. If that's why put it so, you know. Is that is it more of a State government? Or does it sits in a department of communities or a community sort of space?” Participant 06, Male, WA, Health |
| Prioritising of critical infrastructures | “So, there's a lot more focus on those infrastructure damage, basically and rather than events that lead to human health impact. I think in some ways heat as a hazard has gone under the radar a little bit in terms of government policy to address that…” Participant 04, Male, QLD, Climate/Environment |
|  | “Whereas now we understand the true risk, particularly with transport and energy and infrastructure, it's now being approached more broadly than just a health issue, which is very important” Participant 15, Female, QLD, Health. |

Abbreviations: UHI, urban heat island

**Table S4 .** Theme four: The current HVI is faced with methodological inconsistencies, limited understanding, and low confidence among policymakers

| **Sub-themes** | **Supporting quotes (participant)** |
| --- | --- |
| Methodological inconsistencies: standardization | "….there's a risk that there'll be these kinds of products coming from multiple directions using different data sets, different versions of metrics from various places that could get a bit messy and difficult to navigate across the board" Participant 04, Male, QLD, Climate/Environment. |
|  | “I know New South Wales is one of the first ones to have a State level resource and then recently the Australian Climate Service has come out with a national one. Even Griffith recently released one as well. But I think it's a bit of a challenge because we know they don't talk to each other, and they all have kind of slightly different things as well” Participant 15, Female, QLD, Health. |
| Complicated tool: lacks granular details and difficult to understand | “So, you've sort of run the risk of providing it an interpretation of the data, so what does it mean, I think it's really the hardest part” Participant 08, Female, VIC, Emergency Services |
|  | “It is actually quite difficult to interpret the maps sometimes, so you really need the expert people in the room talking you through some of it particularly when it's got various components to it, like you've got the vulnerability risk, and then you've got the coping capacity” Participant 12, Female, ACT, Meteorology. |
| Low level of trust among policymakers | “So, it's not so much the quality of the research, It’s the quality of the interactions between these communities, because the policy makers are not confident in how they should use the data” Participant 03, Male, SA, Meteorology |
|  | “So, if you expect a spike in crime or a spike in ambulance turnouts or hospitalizations are your observations matching what you expected to happen?. I think, until you can kind of test it in theory , it's very difficult to have confidence in in a model” Participant 16, Male, QLD, Emergency Services. |
| Volatility of the input data | “Once you get into a vulnerability map, it's got all those built social natural factors in there that are also going to change over time, so it just makes it very challenging from the climate longer term climate perspective” Participant 04, Male, QLD, Climate/Environment. |
|  | “What I think is that the heat vulnerability indices are going to change rapidly as infrastructure changes, as populations change, as immigrants arrive. So, it’s something that needs to be able to be dynamically refreshed on a fairly rapid update as an asset so that planners can see where risk is growing and diminishing across communities, whether by location or by type of community” Participant 03, Male, SA, Meteorology. |

**Table S5.**Theme five: HMP are well-positioned to evaluate HVI and encourage broader engagement of the index in Australia

| **Subthemes** | **Supporting quotes (participant)** |
| --- | --- |
| Requires validation with health data | “You look at the data, including hospital admissions just to see what's happening is there evidence that the index that you've developed does it track? high vulnerability equal higher number of admissions, that sort of thing" Participant 05, Male, WA, Climate/Environment |
|  | “I would certainly encourage that any of these mapping products should have some sort of evaluation process where you want to look at things like that, absolutely, there needs to be some sort of effort involved in saying that this is then reflected on the ground in terms of how the local community responds to, or impacted by a heat event” Participant 04, Male, QLD, Climate/Environment. |
| Development of the tool requires multidisciplinary engagement | “You need to bring together the communities that are actually engaged in generating the indices and the responses to those indices. To make sure that people have confidence, and how they should use them” Participant 03, Male, SA, Meteorology. |
|  | “…..It would have to be something that is probably developed in partnership or collaboration with State and Federal governments. So, I would look at it being like a cooperative research opportunity or something like that” Participant 16, Male, QLD, Emergency Services |
| Need for community-specific information | “I think one of the reasons is type of people who are the vulnerable people might differ in each city. So, the vulnerable people in Darwin are very different to say homeless people in Sydney or Melbourne they got completely different, like the typical circumstances that they're in are just so different to the type of vulnerable people in Darwin, and I don’t think you can use the same criteria” Participant 13, Female, NT, City Council |
|  | “So, if for instance, somebody is developing a heat vulnerability index they will have to look at this specific characteristics of each community and take that into consideration, if you lump up everything, it wouldn't be useful” Participant 02, Male, NT, Health |
